# Is the dominant leg also the stronger leg in vertical and horizontal jump performance in young male soccer players?

**DOI:** 10.3389/fspor.2026.1785376

**Published:** 2026-03-25

**Authors:** Juan Rabal-Pelay, Rafael Albalad-Aiguabella, José Luis Arjol-Serrano, Demetrio Lozano, Alberto Roso-Moliner, Elena Mainer-Pardos

**Affiliations:** Health Sciences Faculty, University of San Jorge, Villanueva de Gállego, Zaragoza, Spain

**Keywords:** jump performance, leg dominance, soccer academy, soccer footedness, strength asymmetry

## Abstract

**Background:**

Unilateral actions are important in soccer and may create functional differences between the lower limbs. It is unclear whether the dominant leg, usually defined as the preferred kicking leg, corresponds to the stronger limb in unilateral jump tasks. This study aimed to examine the association between dominant leg preference and the strongest leg, and to compare jump performance between dominant and non-dominant legs across age groups in young male soccer players.

**Methods:**

One hundred and sixty-one male soccer players (16.13 ± 1.38 years) from the same soccer academy participated in this cross-sectional study. Players were classified into four age groups (U14, U16, U18, and U19). Unilateral performance was assessed using the single-leg countermovement jump (CMJ) and the single-leg horizontal jump (HJ). Leg dominance was identified based on the preferred limb for soccer-specific actions. Associations between dominant and strongest leg were analyzed using chi-square tests, and differences between dominant and non-dominant legs were examined using paired comparisons.

**Results:**

Significant associations between dominant leg and strongest leg were found only in isolated cases: for HJ performance in the U14 group (*p* = 0.010) and for CMJ performance in the U18 group (*p* = 0.008). No significant associations were observed in the U16 or U19 groups. Additionally, no significant differences were found between dominant and non-dominant legs in unilateral CMJ or HJ performance across any age group.

**Discussion:**

Dominant leg preference does not consistently reflect superior unilateral jump performance in youth soccer players. Isolated associations were observed in specific age groups (U14 horizontal jump, U18 vertical jump), but overall performance was similar between dominant and non-dominant legs. These findings emphasize the task-specific and individual nature of unilateral performance and suggest that leg dominance should not be assumed as an indicator of greater physical capacity.

## 1 Introduction

Unilateral actions play a central role in soccer performance, as players frequently rely on one limb for specific tasks such as kicking, changing direction, accelerating, and jumping. Consequently, increasing attention has been directed toward understanding unilateral performance characteristics and potential performance differences between the lower limbs in soccer players (1–7). In this context, previous studies have highlighted that lower limbs may assume different functional roles depending on the task performed, particularly in sports characterized by repetitive unilateral actions such as soccer (8–10). However, despite this growing body of literature, limited evidence is available regarding whether the dominant leg, commonly defined as the preferred leg for kicking, consistently corresponds to the limb exhibiting superior unilateral jump performance (11, 12). This relationship remains especially unclear in young soccer players, in whom ongoing physical and neuromuscular development across maturation stages may influence task-specific unilateral performance patterns.

Soccer is a highly demanding cooperation–opposition team sport that involves frequent unilateral actions such as sprinting, accelerating, decelerating, changing direction, and jumping, as well as technical skills like kicking and passing (13–19). Due to the repetitive and task-specific use of each limb, lower extremities may assume different functional roles depending on the demands of the action performed (14, 15). This aspect may be particularly relevant during formative stages, when players are still undergoing physical, neuromuscular, and motor development, and unilateral performance characteristics may vary across tasks and age groups (20). Gaining insight into whether the dominant leg also corresponds to the limb demonstrating superior unilateral performance may provide useful information for practitioners working with youth players, supporting the design of training strategies that consider task-specific and individual performance profiles.

Limb dominance, the preferred leg for kicking, does not necessarily coincide with the limb exhibiting superior strength or power across all physical tasks (21). In soccer, the frequent execution of unilateral actions may lead each limb to be preferentially involved in different task-specific demands, such as passing, shooting, jumping, or change of direction (COD) actions (22, 23). As a result, unilateral performance superiority may vary depending on the task performed rather than being consistently associated with leg dominance. Therefore, examining whether the dominant leg corresponds to the strongest leg in specific unilateral performance tasks becomes particularly relevant in the context of youth player development and performance monitoring.

In addition to the aspects mentioned above, another important consideration is the potential variability in identifying the stronger limb across different physical performance tests (24). Because each test places distinct mechanical and neuromuscular demands on the athlete, the limb demonstrating superior unilateral performance may differ depending on the specific task assessed (24, 25). This task-specific variability highlights the need to examine unilateral performance across multiple assessment modalities, rather than assuming a consistent correspondence between leg dominance and performance superiority. A clearer understanding of these distinctions may contribute to a more accurate interpretation of unilateral performance characteristics and support the development of more targeted and effective training interventions in youth soccer players.

To the best of the authors' knowledge, few studies have investigated whether the dominant leg corresponds to the strongest limb across different physical performance tests. Buśko et al. (11) reported no significant differences in strength and jump performance between the preferred and non-preferred legs in elite Polish soccer players, while Clemente et al. (12) observed that leg dominance influenced performance in the 505 COD test, although no such association was found in the 90° COD test or the cross-over hop test in young soccer players. Moreover, Clemente et al. (12) reported no significant differences between the dominant and non-dominant legs in COD performance when comparing players in U16 and U18–U19 age groups. Furthermore, limited research has explored how the association between leg dominance and unilateral performance evolves throughout the different stages of biological maturation. Previous studies have primarily focused on describing unilateral performance differences across age categories in male and female soccer players (1, 3, 26, 27), rather than examining the correspondence between leg dominance and task-specific performance superiority. Therefore, to date, no study has specifically analyzed whether leg dominance corresponds to the strongest limb across multiple unilateral jump tests.

Therefore, the aims of the present study were: (1) to examine the association between dominant leg preference and the strongest leg in unilateral vertical (countermovement jump, CMJ) and horizontal jump (HJ) performance in young male soccer players, and (2) to compare unilateral jump performance between the dominant and non-dominant legs across different age groups (U14, U16, U18, and U19). It was hypothesized that dominant leg preference would not consistently correspond to the limb demonstrating superior unilateral jump performance. Additionally, it was expected that differences in unilateral jump performance between the dominant and non-dominant legs would be small across age groups.

## 2 Methods

### 2.1 Subjects

A total of 161 male soccer players (age: 16.13 ± 1.38 years; height: 176.6 ± 6.71 cm; body mass: 65.3 ± 8.01 kg), all belonging to the same soccer club, voluntarily took part in this study. Players were distributed across four competitive categories (U14, U16, U18, and U19), all of which competed in regional or national federate leagues. Player level was classified according to the Participant Classification Framework proposed by McKay et al. (28), with U19 players categorized as TIER 3 (Highly Trained/National Level) and the remaining players as TIER 2 (Trained/Developmental).

Players with injuries or illnesses in the three weeks prior to the assessments were excluded. All participants (or their legal guardians in the case of minors) provided written informed consent.

All players had accumulated at least three years of training experience with their respective teams, participating in three to four structured technical and tactical sessions per week, each lasting approximately 90 min, in addition to one official match per week. Physical preparation programs varied slightly between teams but typically included drills focused on speed, agility, and quickness, along with exercises aimed at injury prevention and coordination. These sessions were planned and supervised by certified strength and conditioning coaches or similarly qualified professionals. Their main objective was to maintain and improve the players' physical capacities in line with the demands of competitive soccer.

This cross-sectional descriptive study was conducted as part of a collaborative agreement between a Spanish soccer club and San Jorge University. The research protocol was approved by the Clinical Research Ethics Committee of San Jorge University (Ref. 7/1/24-25), and all procedures were carried out in accordance with the principles of the Declaration of Helsinki.

### 2.2 Procedures

All testing procedures were conducted in the players' regular training facility, on a hard, non-slip surface, and under stable environmental conditions (temperature between 21 and 23 °C). Sessions took place between 17:00 and 18:30 to align with the athletes' habitual training schedule and to minimize circadian influences on performance (29). Each participant wore their usual indoor training footwear and sports clothing, consistent with what they typically used during gym-based sessions.

To reduce the risk of fatigue-induced performance alterations, participants were instructed to refrain from engaging in high-intensity physical activity during the 48 h preceding the assessment. They were also advised to avoid the consumption of caffeine or other stimulants, including energy drinks and dietary supplements, that could potentially affect physiological responses. In addition, players were reminded of the importance of maintaining adequate hydration and nutritional intake during this preparatory period to ensure optimal testing conditions.

Prior to testing, athletes completed a structured warm-up lasting approximately 15 min, following the Raise, Activate, Mobilise, and Potentiate model (30). This included light jogging, dynamic mobility work, muscle activation exercises, and progressive jump efforts ranging from submaximal (80%) to maximal (100%) intensity.

All players were already familiar with the jumping tests, having completed them on several prior occasions as part of their regular performance monitoring, thus minimizing potential learning effects. The assessment comprised two unilateral jump tests: the single-leg CMJ and the single-leg HJ. Testing began with the CMJ using the left leg, followed by the right leg. Subsequently, the same order was followed for the HJ: left leg and then right leg. The fixed testing order (left leg first) was used to standardize the testing procedure across all participants and ensure consistency during data collection. For each attempt, participants were required to land on the same foot used for take-off and maintain balance with that foot in contact with the ground for at least two seconds. Two valid attempts were recorded for each leg and jump type, with the best value retained for analysis. A recovery period of 45 s was provided between individual jumps, and a 10-minute rest interval was observed between the two jump modalities. These recovery periods were selected to minimize fatigue effects and allow adequate recovery between maximal unilateral efforts.

#### 2.2.1 Unilateral countermovement jump test

The single-leg CMJ was employed to evaluate vertical power output. Players initiated the movement standing on one leg, keeping their hands firmly on their hips throughout the action. The non-jumping leg was held at approximately 60° of hip flexion and allowed a slight swing to assist with the propulsion phase. Participants performed a quick downward movement by flexing the ankle, knee, and hip joints, immediately followed by an explosive extension aiming to achieve maximum vertical height. During flight, the jumping leg was required to remain extended. Standardized verbal instructions were given to all participants to reduce variability in force–time application and to ensure technical consistency. Landing had to occur in the same take-off spot, and athletes were required to stabilize on the landing foot for a minimum of two seconds to consider the attempt valid. Vertical jump height (cm) was measured using the MyJump mobile application, which provides valid and reliable estimates of jump height via high-speed video capture. Specifically, MyJump has demonstrated high validity compared with force platforms, showing very strong correlations and trivial systematic bias (31). This test has shown excellent reliability in youth soccer populations, with intraclass correlation coefficients (ICC) ranging from 0.90 to 0.96, and coefficients of variation (CV) between 3.1% and 4.8%.

#### 2.2.2 Unilateral horizontal jump test

The single-leg HJ was used to assess forward explosive power in a horizontal plane (32). Athletes initiated the movement from a stable unipedal stance, similar to the CMJ setup, with the non-jumping leg allowed a slight backward swing to support the propulsion phase. Participants were instructed to perform a maximal HJ, taking off and landing with the same leg. Upon landing, they were required to maintain their balance for a minimum of two seconds, without using the opposite foot or making any additional steps or hops. This ensured consistency in the stability requirement across all attempts. The distance achieved was measured in centimeters, from the starting line (toe at take-off) to the heel of the landing foot. A flexible measuring tape placed along the ground was used for this purpose. This test has demonstrated high levels of reliability in youth athletic populations, with ICC reported between 0.88 and 0.95, and CV ranging from 2.1% to 3.5%.

#### 2.2.3 Definition of dominant and Non-dominant Leg

To determine limb dominance, each player was asked to indicate their preferred leg for performing soccer-specific actions such as kicking, passing, or shooting. The leg identified for these tasks was recorded as the dominant leg, while the contralateral limb was classified as the non-dominant leg. This self-report method is widely accepted and commonly used in studies involving soccer players (33). Based on this classification, the sample was divided into two groups: players with right-leg dominance (*n* = 124) and those with left-leg dominance (*n* = 37). This distinction was later used to explore potential relationships between functional dominance and performance outcomes in the jump tests.

#### 2.2.4 Definition of strong and weak Leg

The strong leg was defined as the limb that achieved the highest performance in each specific jump test—either in terms of vertical height (CMJ) or horizontal distance (HJ). Conversely, the weak leg was identified as the one that produced the lower value in the respective test. This classification was applied independently for each type of jump, as previous studies have shown that the dominant limb in one movement may not necessarily be the superior performer in another (21, 24).

#### 2.2.5 Statistical analysis

Statistical analyses were performed using SPSS software (version 29, IBM Corp., Armonk, NY, USA). Continuous variables were described using mean and standard deviation (x̅ ± SD) for each age group and limb. Test–retest reliability of unilateral CMJ and HJ was assessed using the ICC and CV. Data normality was evaluated using the Kolmogorov–Smirnov test. A significance level of *p* < 0.05 was established for all analyses. The association between dominant leg preference and the strongest leg was examined using Pearson's chi-square test. When expected cell frequencies were small, Yates' continuity correction was applied. Effect sizes for chi-square analyses were reported using Cramer's V. Differences in unilateral jump performance between the dominant and non-dominant legs were assessed using paired-samples t-tests. When normality assumptions were not met, the Wilcoxon signed-rank test was used. Mean differences (*Δ* = dominant−non-dominant) and corresponding 95% confidence intervals were reported to support interpretation.

## 3 Results

The means and standard deviations of unilateral CMJ and HJ performance for the dominant and non-dominant legs across age groups are presented in Table 1.

**Table 1:** Descriptive statistics by age group.

Age group (*n*) Dominant leg distribution (%)	Variable	x̅ ± SD (Dominant)	x̅ ± SD (Non-dominant)
U14 (*n* = 21) R: 78.6%/L: 21.4%	CMJ	18.8 ± 2.73	19.4 ± 3.62
	HJ	173.5 ± 12.5	176.1 ± 13.8
U16 (*n* = 56) R: 74.4%/L: 25.6%	CMJ	19.3 ± 2.33	19.4 ± 2.76
	HJ	165.5 ± 14.9	163.5 ± 16.5
U18 (*n* = 41) R: 77.4%/L: 22.6%	CMJ	20.7 ± 3.04	21.2 ± 2.97
	HJ	175.3 ± 10.9	175.9 ± 10.6
U19 (*n* = 26) R: 78.6%/L: 21.4%	CMJ	22.7 ± 3.86	23.1 ± 4.02
	HJ	189.5 ± 10.7	190.1 ± 12.8

CMJ, countermovement jump; HJ, horizontal jump; R, right; L, left.

**Figure 1:**
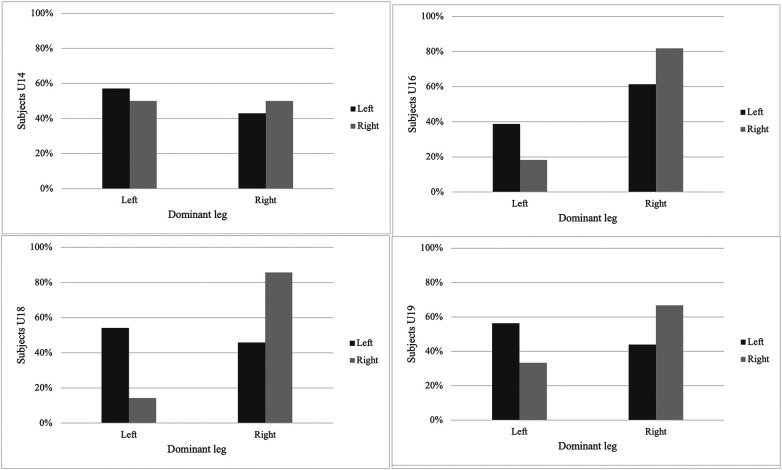
Relationship between dominant leg and strong CMJ by age groups. CMJ, countermovement jump.

**Figure 2:**
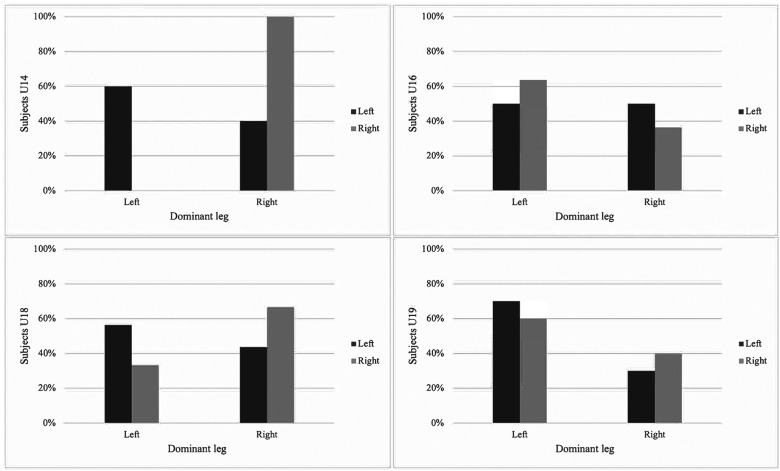
Relationship between dominant leg and strong HJ by age groups. HJ, horizontal jump.

Figures 1, 2 illustrate the percentage distribution of the strongest leg according to dominant leg preference across age groups for unilateral jump performance.

The associations between dominant leg and strongest leg, as well as comparisons between dominant and non-dominant unilateral jump performance, are presented in Table 2. A significant association between dominant leg and strongest leg was observed for HJ performance in the U14 group (*χ*^2^ = 6.68, *p* = 0.010, *V* = 0.51) and for CMJ performance in the U18 group (*χ*^2^ = 6.96, *p* = 0.008, *V* = 0.34). No other significant associations between dominant leg and strongest leg were found across age groups or jump modalities (all *p* > 0.05).

**Table 2:** Association between dominant leg and strongest leg, and dominant vs. non-dominant unilateral jump performance by age group.

Age	Dominant vs. strong leg (*χ*^2^, *p*, *V*)	Dominant vs. non-dominant performance [*p*, *Δ* (95%CI)]
U14	CMJ: 0.10, 0.746, 0.06	CMJ: 0.258, −0.55 cm (−1.52; 0.42)
	HJ: 6.68, 0.010*, 0.51	HJ: 0.157, −2.48 cm (−5.98; 1.02)
U16	CMJ: 1.54, 0.215, 0.19	CMJ: 0.665, −0.11 cm (−0.62; 0.40)
	HJ: 0.59, 0.442, 0.12	HJ: 0.200, 1.98 cm (−1.09; 5.04)
U18	CMJ: 6.96, 0.008*, 0.34	CMJ: 0.067, −0.44 cm (−0.91; 0.03)
	HJ: 1.95, 0.162, 0.19	HJ: 0.533, −0.63 cm (−2.62; 1.37)
U19	CMJ: 0.91, 0.338, 0.20	CMJ: 0.361, −0.35 cm (−1.14; 0.43)
	HJ: 0.18, 0.668, 0.09	HJ: 0.674, −0.56 cm (−3.24; 2.13)

CMJ, countermovement jump; HJ, horizontal jump; *Δ*, mean difference calculated as dominant − non-dominant.

* *p* < 0.05.

Comparisons between dominant and non-dominant leg performance revealed no significant differences for unilateral CMJ or HJ performance in any age group (all *p* > 0.05; Table 2). Mean differences between legs were small across all categories, with values ranging from −0.55 to −0.11 cm for CMJ and from −2.48 to 1.98 cm for HJ.

A non-significant trend toward higher CMJ performance in the non-dominant leg was observed in the U18 group (*p* = 0.067), although this did not reach statistical significance.

## 4 Discussion

The present study examined the association between dominant leg preference and the strongest leg in unilateral vertical and HJ performance, as well as differences in unilateral jump performance between dominant and non-dominant legs across age groups in young male soccer players. The main findings indicate that dominant leg preference does not consistently correspond to the limb demonstrating superior unilateral jump performance. Although significant associations between dominant and strongest leg were observed in specific conditions (HJ in U14 and CMJ in U18), these associations were not systematic across age groups or jump modalities. Furthermore, comparisons between dominant and non-dominant legs revealed no statistically significant differences in unilateral CMJ or HJ performance in any age group. Mean differences between limbs were small across all age categories, suggesting that unilateral jump performance was largely comparable between dominant and non-dominant legs. Together, these findings highlight considerable inter-individual variability in the relationship between leg dominance and unilateral jump performance, rather than a uniform or age-dependent pattern.

In relation to the primary aim of the study (1) to examine the association between dominant leg preference and the strongest leg in unilateral vertical (CMJ) and HJ performance in young male soccer players. The findings revealed no association between the preferred dominant leg and the limb demonstrating superior performance in the U16 and U19 groups. These results indicate that, in mid- to late-adolescent players, self-reported dominance does not systematically correspond to the leg displaying higher unilateral jump capacity. However, a significant association was identified in the U14 group for HJ performance and in the U18 group for CMJ performance. However, it is important to note that this association observed in the U14 and U18 groups was present in only one of the jump tests analyzed. Despite this, these findings support the initial hypothesis and reinforce the notion that leg dominance does not consistently reflect the strongest limb in unilateral jumping actions.

In line with the findings of the present study, Carcia et al. (34) found that the preferred kicking leg (dominant leg) was not necessarily the stronger one in unilateral drop jumps in active students. On the other hand, Hernández-Davó et al. (35) analyzed young tennis players, observing that the dominant leg performed better in the horizontal triple hop test, but reported greater vertical CMJ performance in the non-dominant leg. Clemente et al. (12) reported that a larger proportion of participants exhibited superior performance when executing the cross-over hop test using the dominant leg, and when braking with the dominant leg during the 505 COD test. Conversely, in the 90° COD test, a greater number of individuals achieved better results when performing the directional turn toward the non-dominant leg. Despite this, only in the case of the 505 COD test were these significant. In line with the present study, there appears to be no direct association between the dominant leg and the stronger leg in horizontal jumping or COD actions.

Regarding the second objective of the study (2), the hypothesis was confirmed, as no differences were observed in vertical or HJ performance between the dominant and non-dominant legs across the different age groups. Sánchez-Sánchez et al. (36) also found that young soccer players showed similar unilateral countermovement and non-countermovement jump values between the dominant and non-dominant legs. In the review by McGrath et al. (37), no statistical effects were observed between the dominant leg and unilateral horizontal or vertical jump performance in an adult population. Also, It has been observed in non-athlete adolescents that balance is similar between the dominant and non-dominant legs (38). In accordance with the results of this investigation, in basketball players aged 14 to 18 have not identified a clear relationship between leg dominance and superior performance in the unilateral vertical CMJ (39). In other sports such as climbing and badminton, differences in strength between the dominant and non-dominant upper limbs have been observed, likely because of functional adaptations (40, 41). Delang et al. (33) reported similar isokinetic strength in the quadriceps and hamstrings between the dominant and non-dominant legs in soccer players, regardless of gender and age.

In contrast, in the research conducted by Filipa-Silva et al. (42) with soccer players, the authors observed that the non-preferred leg demonstrated superior acceleration and deceleration performance during a change-of-direction task compared to the preferred leg. Some investigations conducted on resistance-trained males have reported that the dominant leg (identified as the leg preferred for kicking a ball) exhibits greater strength in unilateral exercises such as the single-leg press or the Bulgarian split squat (43, 44). Future studies could analyze performance in strength tests with different expressions (jumps, isokinetic tests, unilateral load lifts…) to clarify the relationship between the dominant leg and the stronger leg in youth soccer players.

The results of our study demonstrate that unilateral jump performance values in vertical (CMJ) and horizontal (HJ) vector increase with age. These findings are consistent with previous research conducted on youth soccer players, which has similarly reported that older athletes tend to achieve better outcomes in jump performance tests (45, 46). This progression could be attributed to biological growth and development, as well as improvements in sports-specific skills acquired through training and experience (47). These results are similar to those reported in young tennis players, showing differences based on maturity status (48). However, in the present study it remains unknown whether this is due to an age-related chronological factor or to potential differences in the groups' maturational status.

One of the main limitations of the study is the lack of assessment of the players' biological maturation. Chronological age and biological maturation may differ within the analyzed age groups (U14–U19). Grouping athletes solely by chronological age may include individuals who are at very different biological stages. This aspect should be considered in future research. A limitation of the study is the lack of information regarding the participants' training exposure and their years of experience in soccer-specific training. Another limitation of the study is that the analyzed population comprised only male players. It is possible that, due to sex-based differences in physical and neuromuscular development during adolescence, the findings may not be generalizable to young female soccer players. Moreover, this cross-sectional research only reflects the jump tests performance levels at a specific point in the season; therefore, the results could change following a training period. On the other hand, players' field positions were not considered (49). Analyzing performance by position and age could reveal relevant information regarding vertical and HJ performance. This study examined the manifestation of explosive strength through jumping. The association between the dominant and strong legs may differ when assessing strength through isometric or dynamic tests (50). Jump performance (jump height) was assessed using the MyJump app; therefore, the use of force platforms could provide additional information on metrics such as impulse, peak force, or the eccentric phase during the unilateral vertical jump. Further research should examine whether the associations reported in this study are similar in the female population and in other higher-performance groups. They should focus also monitor a group of young players over several seasons.

## 5 Conclusions

The present study highlights that there are no age-related differences in the relationship between leg dominance and the stronger leg in unilateral jump performance among youth soccer players. However, significant associations were observed between the dominant and stronger leg in the vertical jump for the U18 group and in the HJ for the U14 group. These isolated associations were not observed in the remaining age groups.

No jump performance differences were found between dominant and non-dominant legs in U14, U16, U18 and U19 age groups. These results indicate that unilateral vertical and HJ performance is similar between the dominant and non-dominant leg across the analyzed age group.

Future research should consider the maturational status of youth soccer players when examining the association between leg dominance and unilateral jump performance. Monitoring these aspects throughout the sporting development of youth soccer players could provide valuable insights into potential changes in jump performance between the dominant and non-dominant leg. This information would be highly valuable for individualizing training programs for young soccer players.

## Data Availability

The datasets presented in this study can be found in online repositories. The names of the repository/repositories and accession number(s) can be found in the article/Supplementary Material.
